# Evaluation of Automated Ribosomal Intergenic Spacer Analysis for Bacterial Fingerprinting of Rumen Microbiome Compared to Pyrosequencing Technology

**DOI:** 10.3390/pathogens3010109

**Published:** 2014-01-22

**Authors:** Elie Jami, Naama Shterzer, Itzhak Mizrahi

**Affiliations:** 1 Department of Ruminant Science, Institute of Animal Sciences, Agricultural Research Organization, Volcani Center, PO Box 6, Bet Dagan 50250, Israel; E-Mails: eliejamie@gmail.com (E.J.); naamash@volcani.agri.gov.il (N.S.); 2 Department of Molecular Microbiology and Biotechnology , The George S. Wise Faculty of Life Science, Tel Aviv University, Ramat-Aviv 69978, Israel

**Keywords:** ARISA, pyrosequencing, rumen microbiome

## Abstract

The mammalian gut houses a complex microbial community which is believed to play a significant role in host physiology. In recent years, several microbial community analysis methods have been implemented to study the whole gut microbial environment, in contrast to classical microbiological methods focusing on bacteria which can be cultivated. One of these is automated ribosomal intergenic spacer analysis (ARISA), an inexpensive and popular way of analyzing bacterial diversity and community fingerprinting in ecological samples. ARISA uses the natural variability in length of the DNA fragment found between the 16S and 23S genes in different bacterial lineages to infer diversity. This method is now being supplanted by affordable next-generation sequencing technologies that can also simultaneously annotate operational taxonomic units for taxonomic identification. We compared ARISA and pyrosequencing of samples from the rumen microbiome of cows, previously sampled at different stages of development and varying in microbial complexity using several ecological parameters. We revealed close agreement between ARISA and pyrosequencing outputs, especially in their ability to discriminate samples from different ecological niches. In contrast, the ARISA method seemed to underestimate sample richness. The good performance of the relatively inexpensive ARISA makes it relevant for straightforward use in bacterial fingerprinting analysis as well as for quick cross-validation of pyrosequencing data.

## 1. Introduction

Analysis of bacterial diversity in environmental samples has always been a challenge for microbiologists. This field has been around for decades—ever since microscopy and culture-isolation methods were first implemented—but accurate assessment of the composition of an environmental sample has been, until recently, limited by our inability to culture most of the bacteria found in a given environment [1]. In the last decade however, with the improvement of high-throughput technologies and bioinformatic tools for analysis, many methods for the characterization of microbial diversity in complex environments have emerged. One such popular method, introduced by Fisher and Triplett (1999) [2], is automated ribosomal intergenic spacer analysis (ARISA). This method amplifies the DNA fragment found between the 16S and 23S genes in bacterial genomes and uses its natural variability in length—and separation by a capillary electrophoresis system (such as Sanger sequencing technology)—to infer diversity, with different sizes representing different operational taxonomic units (OTUs). This method is thought to describe the bacterial community at species level resolution [3], but has been shown to have some limitations in terms of accurately depicting microbial diversity in samples. A study by Kovacs *et al.* (2010) [3] revealed that with increasing species diversity, the method tends to underestimate species richness. This is the result of the limited fragment lengths that could be detected by this method, which range from 200 to 1,150 bp, restricting the number of different observable phylotypes within a sample to several hundreds. This means that ARISA might not be the most adequate assessment method for comparisons between samples with high taxon richness [4,5,6,7]. Nevertheless, ARISA has been extensively used for bacterial community analyses along with other non-sequencing methods such as DGGE or TRFLP [8,9,10,11,12,13], and a comparative study have found that it performed better than the other mentioned methods [9]. Another important aspect of community analysis lies in the ability of the fingerprinting method to accurately discriminate between samples for comparative analyses between different environments or conditions. In this context, ARISA was used to detect changes in bacterial community composition of a highly complex microbial environment residing in the rumen—the upper digestive tract compartment found in all ruminants [14,15,16,17]. Despite all of its benefits, ARISA falls short in comparison to the robust and now more accessible sequencing technologies. The latter’s only limitation is the depth in which the samples are sequenced, thus it is less prone to underestimate sample richness. Additionally, pyrosequencing provides taxonomic identification by making use of the very comprehensive 16S rRNA databases available online [18]. Although the pyrosequencing methods are dropping in price, they remain more expensive than the fingerprinting methods. Furthermore, microbial ecology studies usually do not have an intrinsic reference point for control of the method itself [19]. Therefore, due to the different approach used with ARISA for microbial community analyses, this method, and its other fingerprinting counterpart could provide a quick and cost effective cross-validation procedure for pyrosequencing results, as previously reported [10,20]. Given its reported limitation with highly complex samples, in this study we aimed to systematically compare ARISA to pyrosequencing in rumen samples from cows at different ages, containing different levels of complexity and exhibiting high and low α-diversity estimates, as previously observed by pyrosequencing [21]. We assessed the degree of agreement between the two methods, in terms of their ability to describe bacterial diversity and correctly discriminate between the sampling groups.

## 2. Comparison of Local Richness Obtained from ARISA *vs.* Pyrosequencing

We collected the rumen contents of 21 animals from different age groups: 1–3-day-old calves (three 1-day-olds and three 3-day-olds, n = 6), 2-month-old calves (n = 5), 6-month-old heifers (n = 5) and 24-month-old lactating dairy cows (n = 5). These groups were fed according to conventional husbandry feeding programs for each age at our facility. Microbial cells were separated and metagenomic DNA was extracted as previously described [22,23]. We then used both bacterial tag-encoded amplicon pyrosequencing generated from the V2 and V3 regions of the 16S rRNA gene and ARISA to characterize the overall bacterial diversity in each of our samples. These samples were used in a previous study for the characterization of rumen bacterial composition across different ages [21]. In that study, the α-diversity in the samples was found to increase with the age of the animal [21], thereby enabling a comparison of the ecological estimates of ARISA with those of pyrosequencing in samples with different complexities. As we previously reported, after size-filtering, quality control and chimera removal using the QIIME pipeline [24], a total of 227,414 quality reads were generated from the pyrosequencing effort with an average of 10,800 ± 2,860 reads per sample. The overall number of OTUs detected by the pyrosequencing analysis was 6,594 based on ≥97% nucleotide-sequence identity between reads. The total number of OTUs associated with each age group, and their respective average OTU numbers per sample are reported in Table 1. Note that the samples from 2-year-old and 6-month-old animals exhibited different total possible OTU numbers and significant differences in OTU number per sample (*P* < 0.05 using *t*-test analysis). Whole-community assessment using ARISA was performed using GeneMarker (SoftGenetics, USA) for ARISA resolution and noise filtering as previously carried out in previous studies [2,16]. The analysis revealed an overall lower bacterial diversity and a significantly lower average OTU number per sample as compared to the pyrosequencing (Table 1), with a total of 341 OTUs detected by ARISA. ARISA showed statistically significant discrimination between each age group—except between the 6-month-old and 2-year-old samples—when OTU numbers where compared, whereas the pyrosequencing method discriminated between all groups based on the same parameter (Table 1, *P* < 0.05 using *t*-test). However, Shannon diversity was significantly different between each group using the pyrosequencing data, whereas the ARISA data only discriminated between the 1–3-day-old samples and the rest of the samples, but not between the older age groups.

## 3. β-Diversity Calculation

**Figure 1:**
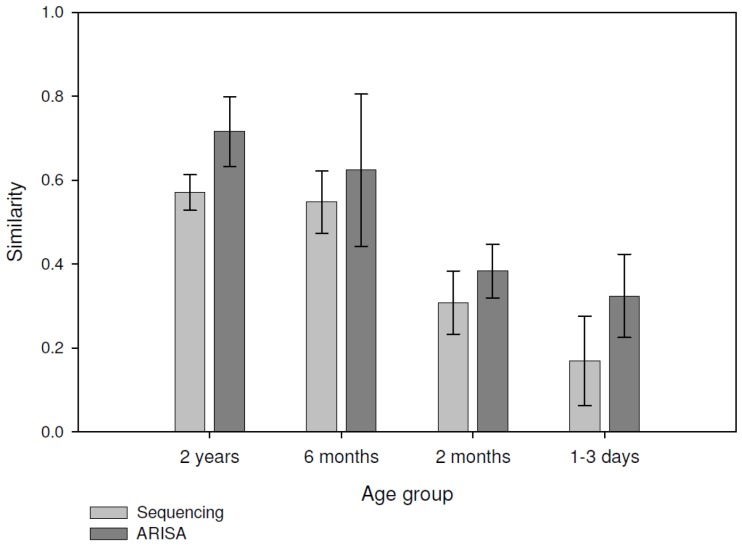
Average Bray-Curtis similarity comparison between ARISA and pyrosequencing for each age group. Light-gray bars: pyrosequencing-based similarity values; dark-gray bars: ARISA-based similarity values.

**Figure 2:**
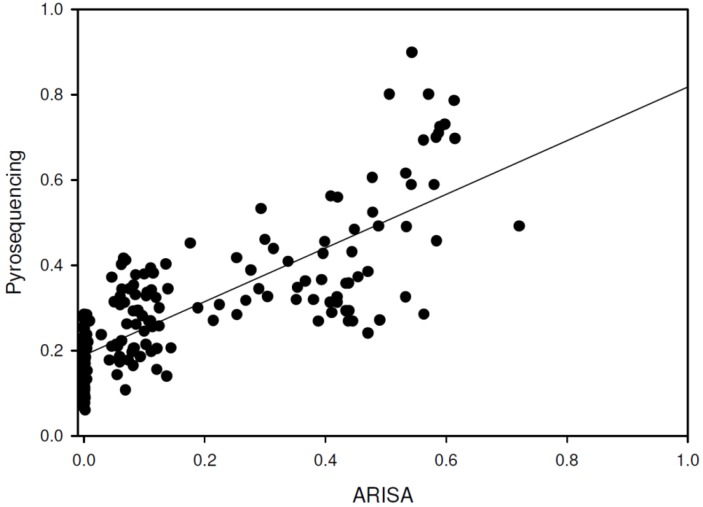
Regression plot for each similarity measurement. Each point represents the similarity comparison between two samples from sequencing (Y-axis) and ARISA (X-axis). The R^2^ for the plot is 0.6.

**Figure 3:**
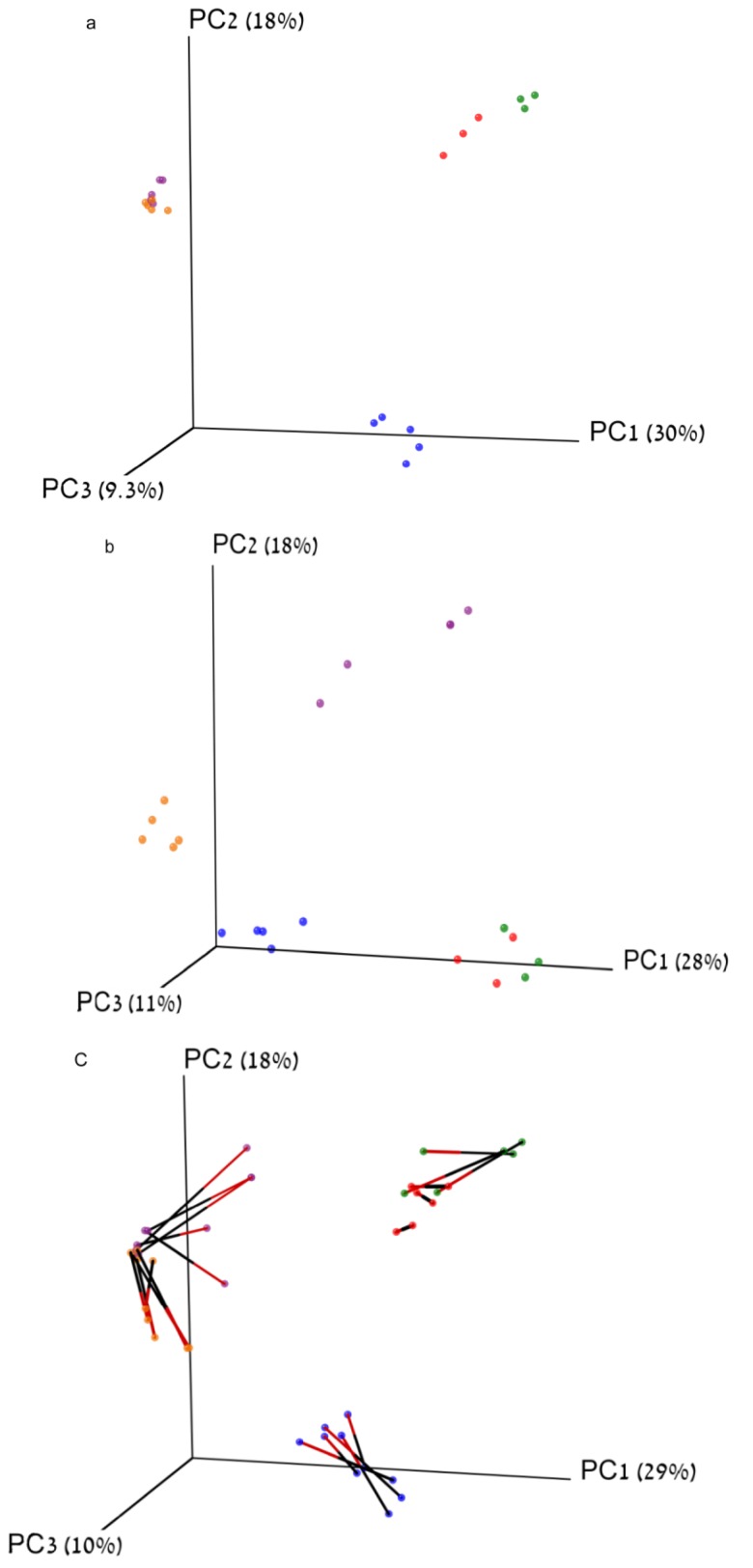
Distance ordination. Principal coordinate analysis (PCoA) plots were generated using the pairwise distance values for each sample using the Bray-Curtis metric. Every point in the plots represents the community in a single sample from either 16S pyrosequencing or ARISA data and is colored according to the animal’s age group (red, 1 day old; green, 3 days old; blue, 2 months old; orange, 6 months old, and purple, 2 years old). **(a)** PCoA of the distances resulting from the sequencing data. **(b)** PCoA of the distances resulting from the ARISA data. **(c)** Procrustes transformation analysis of 16S rRNA sequences against the ARISA-based operational taxonomic unit (OTU) clustering. The orange end of each line connects to the 16S rRNA data for the sample, and the black end of the line is connected to ARISA data for the sample.

The pairwise similarity within each group was calculated using the Bray-Curtis index, calculated from both the pyrosequencing and ARISA datasets, and the statistical significance of the changes in average similarity between groups was calculated as well (Figure 1). The ARISA average similarity values were consistently higher than those based on pyrosequencing for all groups (*P* < 0.05), However there was a strong correlation between ARISA and pyrosequencing values for each sample similarity (Figure 2; Pearson R = 0.8, *P* < 0.001). We used analysis of similarity (ANOSIM) to assess whether the groups are indeed separate from one another using the pairwise similarity values. For both pyrosequencing and ARISA, all of the groups were significantly different from each other (Table 2, Table 3). Each distance matrix of the samples for both ARISA and pyrosequencing were plotted using principal coordinate analysis (PCoA) (Figure 3a, b). The two methods clearly showed age group-based clustering with the two older groups clustering closer together, while the 2-month-olds and 1–3-day-olds clustered only within their own group. ANOSIM for both methods showed that the groups were indeed distinct from one another for both the pyrosequencing and ARISA data (Table 2 and Table 3). Interestingly, the ARISA data separated the samples taken from 6-month-olds and those taken from 2-year-olds more clearly. Using Procrustes transformation, available in the QIIME package [24], two different PCoAs of the same samples can be superimposed by using different sets of data for analysis, and the degree of agreement between the two different sets of information for the same samples can be compared [25]. The transformation was performed on the three-dimensional principal coordinate analysis (PCoA) resulting from the pyrosequencing and ARISA data using Bray-Curtis as the distance index (Figure 3c). The goodness of fit (M^2^) was calculated for the first three dimensions and was 0.45 with *P* < 0.00001 based on 1,000 Monte-Carlo permutations. This revealed that the methods reached strong agreement in the clustering of the samples, allowing similar conclusions to be drawn about the degree of bacterial similarity and diversity between the samples and their respective groups (Figure 3).

## 4. Discussion

Up until the recent rise in the use of next-generation pyrosequencing for bacterial fingerprinting, ARISA was a popular method of studying community diversity in gut samples, and was considered more accurate than other non-sequencing PCR-based community-analysis methods, such as TRFLP or LH-PCR [9,11]. We evaluated the sensitivity and accuracy of ARISA compared to community analysis based on 16S whole-community pyrosequencing. The fact that these samples originated from the gut environment using identical sampling and extraction procedures allowed us to compare the performance of these two methods on various complexity measures using different ecological parameters. The number of OTUs revealed by the ARISA was significantly lower than that obtained with the pyrosequencing analysis based on 97% similarity. In addition, using ARISA there were no statistical differences in OTU numbers or diversity metrics between the more complex samples (6-month-olds and 2-year-olds), making it less sensitive in discriminating richness between different environments or conditions. Pyrosequencing, on the other hand, did reveal a difference between the 2-year-old and 6-month-old samples. This could be explained by the lower sensitivity of the ARISA method, which underestimates richness in extremely rich environments as a result of a limited dynamic range and detection threshold, resulting from noise ratio for fluorescent fingerprinting techniques requiring to discard all signals under 0.1% abundance [3,26]. Additionally, it was previously observed that different bacterial taxa may have similar intergenic spacer length, or that the same taxa might have more than one ITS sequence length, further skewing sample richness [3,27,28]. Consequently, ARISA may not be an appropriate tool for assessing species richness within complex samples compared to the pyrosequencing method. Recent studies showed comparable underestimations of the bacterial community richness when ARISA was compared to pyrosequencing, ranging from 10 to 100 times lower OTU numbers [4,7,29]. However, both the ARISA and pyrosequencing data generated β-diversity estimates significantly discriminated between the different groups tested in a similar manner [7]. This held true in the current study as well in terms of relative diversity and similarity, as reflected by Procrustes analysis, ANOSIM and within-group similarity values (Figure 1, Figure 2, and Table 2, Table 3). In contrast to α-diversity, β-diversity using the Bray-Curtis index managed to differentiate between the high-complexity samples in both methods, using the compositional and abundance differences of the observed OTUs. within this respect, ARISA demonstrated sharper discrimination between the 6-month-old and 2-years-old animals, compared to the higher degree of similarity between the groups observed using pyrosequencing. This highlights the comparable ability of ARISA to discriminate between different environments based on OTU composition despite its lower resolution, as also observed recently in samples of coastal sand bacterial communities [7].

**Table 1:** Number of OTUs observed (OTU ≥ 97% considered species level) and Shannon-Wiener diversity for each age group, using ARISA and pyrosequencing results. Values in the same column with different superscript letters are significantly different (*P* < 0.01).

	Number of OTUs identified (number of OTUs per sample)		Shannon-Wiener (*H'*) diversity (SD)	
	Pyrosequencing	ARISA	Pyrosequencing	ARISA
1–3 days old	380 (208 ± 47 ^a^)	206 (90 ± 18 ^a^)	2.8 (0.49) ^a^	3.7 (0.31) ^a^
2 months old	1441 (620 ± 100 ^b^)	204 (116± 8 ^b^)	3.7 (0.36) ^b^	4.2 (0.15) ^b^
6 months old	4074 (2051 ± 210 ^c^)	235 (141 ± 20 ^c^)	6.2 (0.3) ^c^	4.2 (0.23) ^b^
2 years old	4885 (2382 ± 263 ^d^)	214 (148 ± 23 ^c^)	6.5 (0.08) ^d^	4.4 (0.35) ^b^

**Table 2:** Analysis of similarity between the age groups, based on the pairwise distance between samples, obtained using the pyrosequencing data. The R-values between each group are displayed in the lower left part of the table. The values in bold in the upper right part of the table represent the Bonferroni corrected *P*-values obtained between the groups.

Pyrosequencing	2 years	6 months	2 months	1–3 days
2 years	0	0.043	0.042	0.01
6 months	0.916	0	0.034	0.012
2 months	1	1	0	0.015
1–3 days	1	1	1	0

**Table 3:** Analysis of similarity between the age groups based on the pairwise distance between samples obtained using the ARISA data. The R-values between each group are displayed in the lower left part of the table. The values in bold in the upper right part of the table represent the Bonferroni corrected *P*-values obtained between the groups.

ARISA	2 years	6 months	2 months	1–3 days
2 years	0	0. 047	0. 047	0. 012
6 months	0.96	0	0. 047	0. 013
2 months	0.684	0.948	0	0. 012
1–3 days	0.9387	0.7893	0.984	0

## 5. Experimental Section

### 5.1. Animal Handling and Sampling

The experimental procedures used in this study were approved by the Faculty Animal Policy and Welfare Committee of the Agricultural Research Organization (ARO), approval number IL-168/08, Volcani Research Center, and were in accordance with the guidelines of the Israel Council on Animal Care.

Israeli Holstein Friesian lactating cows and heifers (n = 21) were housed at the ARO’s experimental dairy farm in Bet Dagan, Israel. Pre-weaned calves were housed separately, while adult animals were housed in one shaded corral with free access to water. The cows were fed according to the conventional feeding regimen at our farm [21]. The samples were taken 1 h after the morning feeding: ruminal contents were collected via the cow’s mouth—500 mL from adults and 100 mL from young calves—using a stainless-steel stomach tube with a rumen vacuum sampler. Samples were transferred to CO_2_-containing centrifuge bottles to maintain anaerobic conditions, and kept on ice. After collection, the samples were processed in the laboratory adjacent to the farm.

### 5.2. Isolation of Microbial Fraction from the Rumen

The microbial fraction was isolated according to [22] with the modifications described by Jami and Mizrahi (2012) [17]. Briefly, following 2 min of blender homogenization, we centrifuged the rumen samples at 10,000 *g* for 30 min at 4 °C and dissolved the pellet in extraction buffer (100 mM Tris-HCl, 10 mM ethylenediaminetetraacetic acid [EDTA], 0.15 M NaCl pH 8.0). The suspension was held at 4 °C for 1 h to maximize the release of particle-associated bacteria from the ruminal contents [22]. The suspension was then gently centrifuged at 500 *g* for 15 min at 4 °C to remove ruptured plant particles while keeping the bacterial cells in suspension [30]. The supernatant was filtered through four layers of cheesecloth and centrifuged (10,000 *g*, 25 min, 4 °C), and the pellets were kept at −20 °C until DNA extraction.

### 5.3. DNA Extraction

DNA extraction was performed by bead disruption with phenol, followed by phenol/chloroform DNA extraction as described by [22]. Isopropanol was then used for precipitation (0.6:1 v/v) and the precipitate was resuspended in 50 to 100 μL Tris-EDTA buffer, then stored at 4 °C for short-term use, or archived at −20 °C.

### 5.4. Automated Ribosomal Intergenic Spacer Analysis (ARISA)

DNA from all rumen samples was subjected to PCR amplification for ARISA [2]. The oligonucleotide primers ITSF (5'-GTCGTAACAAGGTAGCCGTA-3') and ITSRtet (5'-GCCAAGGCATCCAAC-3') were used for ARISA of rumen bacteria, with the fluorescent molecule TET used as described recently by [15]. ARISA PCRs were carried out in 15-µL volumes containing Fermentas Dreamtaq (Madison, WI) master mix, 0.5 µL of 10 µM stock solution for each primer, 20 ng of template DNA and 4.5 µL of nuclease-free water. PCR was carried out using a Sensiquest thermocycler (Gottingen, Germany) under the following conditions: 94 °C for 2 min (1 cycle), followed by 30 cycles of 94 °C for 1 min, 55 °C for 60 s and 72 °C for 120 s, and finally 1 cycle at 72 °C for 5 min.

### 5.5. ARISA Resolution and Analysis

For each DNA sample, two technical replicates of PCR products were analyzed using an ABI PRISM 3,100 Genetic Analyzer. The labeled fragments were separated on the capillary sequencer along with a custom-made ROX-labeled 250- to 1,150-bp standard (Bioventures). Raw data generated by the genetic analyzer were initially analyzed using GeneMarker (Softgenetics, USA) according to [3]. After performing accurate size calling using the program, all data were exported to Microsoft Excel for further analysis. All OTUs with fluorescence intensity of ≤10 RFU (relative fluorescence units) were excluded. The remaining OTUs were binned as described by [27] with the following parameters: bins of 3 bp (±1 bp) for fragments up to 700 bp in length, bins of 5 bp for fragments between 700 and 1,000 bp in length, and bins of 10 bp for fragments longer than 1,000 bp. Intensities were then summed for each bin. Next, relative intensities for each binned OUT in a given sample were calculated and OTUs, which constituted less than 0.1% of the total intensity of the sample, were excluded. Technical duplicates were compared as individual samples and served as an internal control for the quality of the analysis. Duplicates that were not similar to each other were further checked for technical-run problems and were either discarded or run again.

### 5.6. 454 Tag Amplicon Pyrosequencing and Data Analyses

454 Amplicon pyrosequencing of the ruminal DNA samples was performed by the Research and Testing Laboratory (Lubbock, TX) using primers covering the 103- to 530-bp region of the 16S rRNA gene sequence which corresponds to the V2 and V3 regions (107F: 5'-GGCGVACGGGTGAGTAA-3' and 530R: 5'-CCGCNGCNGCTGGCAC-3'). The tagging and sequencing protocol was as described by [31]. Data quality control and analyses were mostly performed using the QIIME pipeline [24] and as described by Jami *et al.* (2013). Briefly, reads were assigned to their designated rumen sample, then length-based filtering (<200 bp was excluded from the analysis), read-quality filtering and chimeric-sequence removal [32] were performed. Binning of OTUs according to the predefined threshold of >97% similarity was performed using the Uclust clustering method [33]. Clusters comprising only one (singletons) or two (doubletons) reads were removed.

### 5.7. Statistical Analyses

Procrustes analysis was performed in QIIME, and the two individual three-dimensional principal coordinates (PCoA) (one for each analytical method) were generated based on a distance matrix calculated using the Bray-Curtis index [34], transformed and visualized in a plot. Statistical analyses of the OTU data were performed using PAleontological STatistics (PAST) software [35], including diversity indexes, correlations and ANOSIM.

## 6. Conclusion

ARISA proved to be a reliable tool for the discrimination of samples based on β-diversity data. Nevertheless, it exhibited some limitations in estimating α-diversity in highly complex samples, as compared to the pyrosequencing method. Despite the decreasing cost of pyrosequencing and the increasing demand in taxonomy for community assessments, ARISA remains an accurate and relevant method for comparing different environments and/or different treatments, with the added advantage of still being considerably less expensive than pyrosequencing. Moreover, it can serve as a good cross-validation procedure for pyrosequencing techniques.
